# Transitioning solidification mode via electroplated Ni coatings in martensitic stainless steel resistance spot welds: new insights into fabricating tough microstructure

**DOI:** 10.1038/s41598-024-53897-1

**Published:** 2024-02-12

**Authors:** Hamidreza Aghajani, Milad Bahrami Balajaddeh, Majid Pouranvari

**Affiliations:** 1 Department of Materials Science and Engineering, Sharif University of Technology, Tehran, Iran; 2 Department of Materials Engineering, Tarbiat Modares University, Tehran, Iran

**Keywords:** Welding, Martensitic stainless steels, Electroplating, Toughness, Solidification mode, Microstructure, Mechanical properties, Mechanical properties, Metals and alloys

## Abstract

The present study addresses the enhancement of fracture toughness of martensitic stainless steel (MSS) spot welds by utilizing through electroplating of Ni on MSS sheets. The equilibrium and non-equilibrium solidification modelling showed that by Ni coating with 50 μm thick on 1.5 mm thick MSSs, the solidification mode changes from δ-ferrite to γ-austenite, leading to a weld nugget (WN) dominated by austenite grains. Moreover, electron backscatter diffraction (EBSD) and electron probe microanalysis (EPMA) showed that the other phases (martensite, δ-ferrite) appeared in band areas of WN owing to incomplete mixing of MSS and the Ni-coating. The tough microstructure in the Ni-coated MSS spot welds provided superior mechanical properties compared to non-coated welds, both in cross-tension (CT) and tensile-shear (TS) tests. Notably, the TS and CT strengths of the Ni-coated MSS spot welds showed a remarkable increase of 57% and 127%, respectively, in comparison to the conventional bare MSS spot welds. Furthermore, in terms of failure energy, the Ni-coated MSS spot welds demonstrated a substantial enhancement of 296% in TS and 520% in CT, when compared to their non-coated counterparts. This research study showcased the effectiveness of Ni electroplating as an industrial method for improving the spot weldability of MSSs.

## Introduction

Martensitic stainless steels (MSSs) are promising materials as a suitable alternative for hot stamped steels in the automotive industry, thanks to their combination of strength and corrosion resistance^1–6^. It was reported^7^ that through appropriate heat treatment, the strength of this class of stainless steels can reach up to 2GPa. However, MSSs are difficult-to-weld due to their brittle microstructure in both the weld nugget (WN) and the heat-affected zone (HAZ), resulting in premature fracture at inadequate load^8,9^.

Different pathways have been explored in literature to enhance the weldability of MSSs, including strategies like enlarging weld nugget size^8,10^, in-situ tempering of weld nugget^11,12^, and employing Ni interlayer^9,13^. Attempts to enlarge WN size by increasing the welding current have revealed limited impact on the microstructure. Notably, despite considerable WN enlargement beyond a threshold size, MSSs retain a highly brittle martensitic microstructure in the WN, resulting in negligible mechanical property enhancements^8,10,14^. It has also been shown that the in-situ post-weld heat treatment through the martensite tempering mechanism improves the mechanical properties in tensile-shear (TS); however, the cross-tension (CT) properties did not improve much and just barely met the minimum standard strength criteria^11,12^. Conversely, the utilization of a nickel interlayer has demonstrated substantial improvement in both the TS and CT mechanical properties of AISI420 MSSs by the authors of this study^9,13^, surpassing minimum strength requirements by a considerable margin. It is worth mentioning that various interlayers have been examined for purposes beyond enhancing weld nugget toughness, e.g. modifying intermetallic compounds (IMCs) in dissimilar metal welding^15–20^. Notably, introducing a 0.5 mm thick Ni interlayer between 1.5 mm MSSs led to a notable modification in the chemical composition of WN, resulting in a remarkably tough austenitic microstructure in the WN^9^, a concept that has since been adopted by subsequent research studies^21,22^ to enhance the weldability of steels. Additionally, the thickness of the Ni interlayer has been identified as a critical factor influencing the phase transformations of the WN^13^. In a recent study^13^, the same research group focused on designing an engineered microstructure to enhance the weldability of MSSs in the automotive industry. This was achieved by strategically controlling the solidification mode within the WN by inserting various thicknesses of Ni interlayer between MSS base metals. The investigation revealed that while greater interlayer thickness generally results in better mechanical properties, a significant increase in mechanical properties occurs with a critical thickness of Ni interlayer based on base metal (BM) thickness and welding parameters. This thickness shifts the solidification mode from primary delta ferrite (δ) to austenite (γ), leading to the formation of a predominantly ultra-tough austenitic microstructure in the WN. This innovative concept substantially improved the mechanical properties of spot welds. In the best-case scenario, the CT and TS strength were about 4 times and twice higher than conventional MSS spot welds, respectively, making these welds highly reliable. That idea was able to improve weldability of MSSs more efficiently than the other approaches such as in-situ rapid tempering or simply increasing WN diameter^11–13^. Although there are limitations to implementing Ni interlayer within the automotive industry, the study provided a systematic guideline for selecting a critical interlayer thickness for spot welding of steels. The aim of this paper now is to translate this concept and research findings into practical applications within the automotive industry. In pursuit of this goal, in this study, MSS base metals underwent an electroplating process to deposit a suitable thickness of Ni coating onto them in such a way as to shift the solidification mode from δ to γ, resulting in a predominant γ microstructure within the WN.

## Experimental procedure

### Ni coating of MSS base metals

In this study, as-received annealed and uncoated AISI420 MSS with a 1.5 mm thickness underwent an electroplating process to deposit a 50 μm thickness of Ni layer coating on one side.

**Figure 1:**
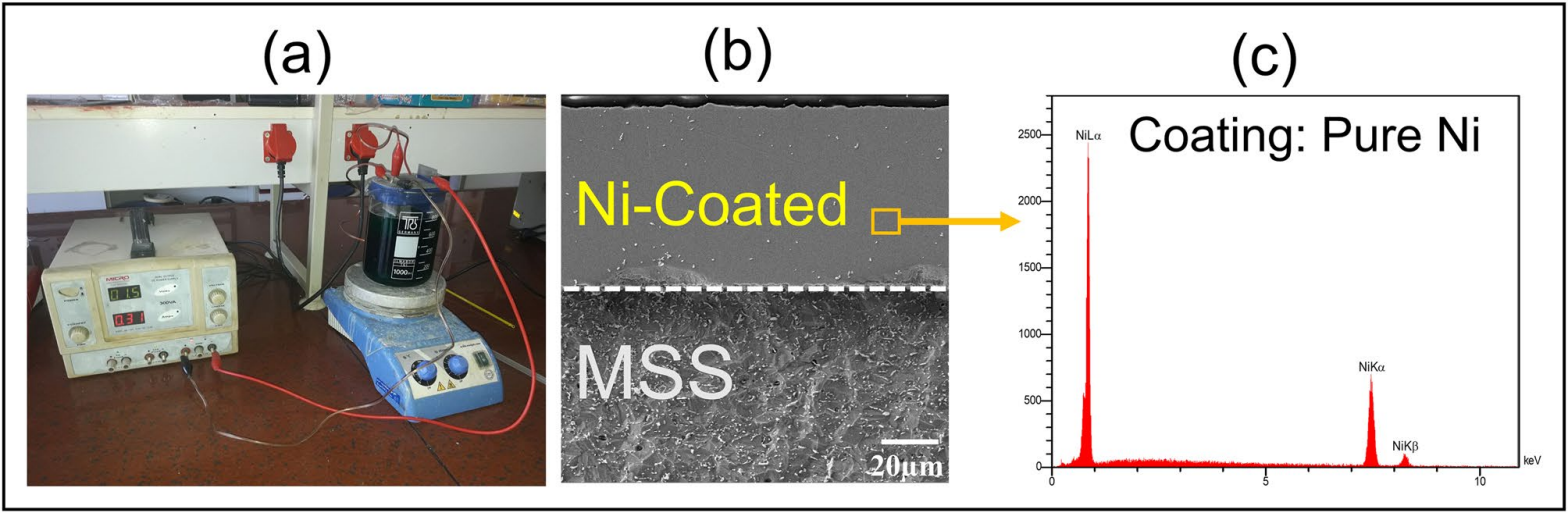
(**a**) The setup for Ni electroplating procedure on MSS sheets, (**b**) the SEM image of the Ni-coated MSS with 50 μm Ni coating on 1.5mm thick MSS, (**c**) EDS map analysis of Ni coating.

Table 1 summarizes the chemical composition and mechanical properties of MSS as the base metal in this study. The electroplating process involved a series of steps to ensure the surface was prepared for optimal coating adherence and quality. First, the desired surface for electroplating was ground and then cleaned and degreased with acetone. Following this, electric acid washing was employed with 20% sulfuric acid by volume at room temperature at a current density of 10.75 amps/dm^2^ for 2 min, with a lead cathode utilized in the setup^23^. The desired surface was then activated in Woods bath (including nickel chloride and hydrochloric acid) at room temperature and a current density of 16.2 amps/ dm^2^ for a duration of 2 min, where the anode was pure nickel. The final stage of the electroplating process involved the deposition of the desired coating onto the prepared surface in a Watts bath^24^, containing nickel sulfate, nickel chloride, boric acid, and sodium dodecyl sulfate. The process utilized a current density of 5 amps/ dm^2^ for a duration of 95 min at a temperature of 50 °C, to achieve a coating thickness of 50 μm at this step. The objective of this electroplating step was to achieve a coating thickness of at least 50 μm. To ensure the desired coating thickness was attained, the electroplating procedure was carried out with specific current density values ranging between 60 and 120 min. Subsequently, cross-sectional samples were obtained using electrical discharge machining (EDM), and the desired thickness was obtained in 95 min. Figure 1a shows the setup for the electroplating procedure. Figure 1b,c illustrates the SEM image of the Ni-coated MSS steel with the EDS map analysis confirming that the coating is pure Ni.

**Table 1:** Properties of as-received uncoated AISI 420 martensitic stainless steel as the base metal in this study.

Material	Chemical composition (wt%)						Mechanical properties		
	Fe	C	Cr	Ni	Mn	Si	YS [MPa]	UTS [MPa]	TEL [%]
AISI420 MSS	Balance	0.18	14.01	0.072	0.3	0.5	290	519	32

### Welding procedure, microstructural and mechanical assessments

After coating the 50 μm Ni on the MSS substrate, the samples were joined with resistance spot welding (RSW) process. The welding parameters included electrode force (5 kN), squeezing time (0.8 s), welding current (9 kA), welding time (0.24 s), and electrode holding time (0.2 s) based on previous experiments^11,13^, to achieve a minimum weld nugget size of 4√t (where t is the thickness of BM) according to AWS standard D8.9^25^. For microstructural observations, standard metallographic procedure (grinding, polishing, and etching) was utilized. The Villela etchant (1 g of picric acid, 5 ml of hydrochloric acid and 100 ml of ethanol) was used to etch the samples. Optical microscopy, field-emission scanning electron microscopy (FE-SEM) equipped with EDS, and electron backscatter diffraction (EBSD) analysis with a step size of 0.15–1 μm were performed to study different microstructure phases, grain evolutions, texture, etc. in the weld nugget. Electron probe microanalysis (EPMA) was employed to analyze quantitative elemental distributions in weld nugget for different spot welds. Vickers microhardness was performed with a 500-gf load and 10 s as dwell time. The mechanical response of spot welds was examined under tensile-shear (TS) and cross-tension (CT) loading conditions with crosshead speed of 5 mm/min and 2 mm/min, respectively. Three repetitions were conducted for each of the uncoated and Ni-coated MSSs under both TS and CT mechanical tests.

## Results and discussion

### Low fracture toughness of uncoated MSS spot welds

The TS and CT strength of bare MSS spot welds (without coating) are 6.5 and 2.2 kN, respectively. It's noteworthy that based on the AWS automotive standard^25^, MSS resistance spot welds with a base metal strength of 520 MPa and a thickness of 1.5 mm must possess minimum strengths of 7 and 3 kN in TS and CT, respectively. However, even MSS spot welds with the recommended weld nugget size (5.8 mm $>$ 4t^0.5^) fail to meet these strength criteria. Moreover, the energy absorption (the area under the load–displacement curve up to the peak load) was measured as 2.5 J for TS and 8.4 J for CT tests. The weak mechanical properties under both TS and CT loading conditions can be attributed to the presence of brittle martensitic microstructure within the WN and HAZ, leading to very low fracture toughness of MSS RSWs, as detailed elsewhere^8,10,11,26^. The microstructure of the WN predominantly consists of martensite, with a presumed presence of residual delta ferrite and a minor amount of austenite^13^. Moreover, the HAZ comprises of martensite and carbides^8,11,13,26^.

### Microstructural observations in Ni-coated MSS spot welds

**Figure 2:**
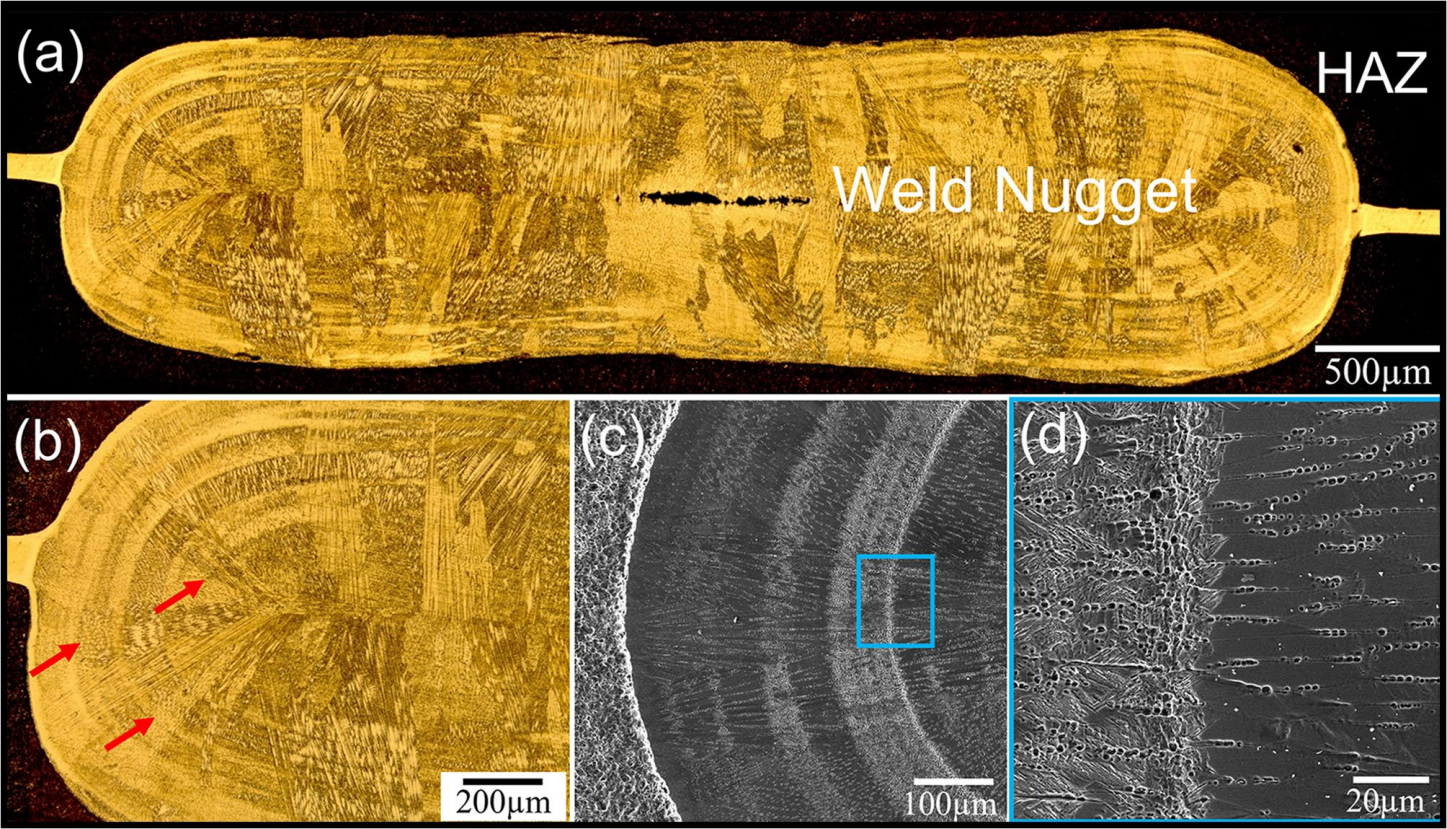
(**a**,**b**) optical micrograph of spot welding of Ni-coated MSSs at different magnifications, (**c**,**d**) SEM images of weld nugget of Ni-coated MSS spot welds, showing the interface of band zones with the rest of weld nugget.

Figure 2a,b illustrates the optical micrograph of the weldment for the coated MSSs with Ni. Evidently, the Fig. 2a highlights the formation of a pore or cavity in the center of the nugget after mixing the melted Ni-coated layer and the base metals during welding. It is believed that the formation of cavities in the weld nugget is attributed to hot tearing phenomenon^27–30^. Hot tearing occurs due to obstructed solidification shrinkage as well as the thermal shrinkage of the weld nugget during the cooling process. The presence of the surrounding base metal connected to the weld nugget results in obstructed shrinkage, generating tensile stresses that ultimately lead to the occurrence of hot tearing in the remaining liquid during the final stage of solidification. In RSW process, these tensile stresses can be mitigated by applying an appropriate electrode force. Therefore, the electrode force and electrode holding time after current-off should be optimized in order to eliminate or minimize the occurrence of cavities within the weld nugget^27^.

Additionally, different areas in the WN have different responses to the etchant, leading to the formation of band areas or onion-ring structure within the WN. Figure 2c,d shows the SEM images at different magnifications of the interface of these areas with the bulk of the WN, which seems to have a different microstructure from the rest of the WN.

**Figure 3:**
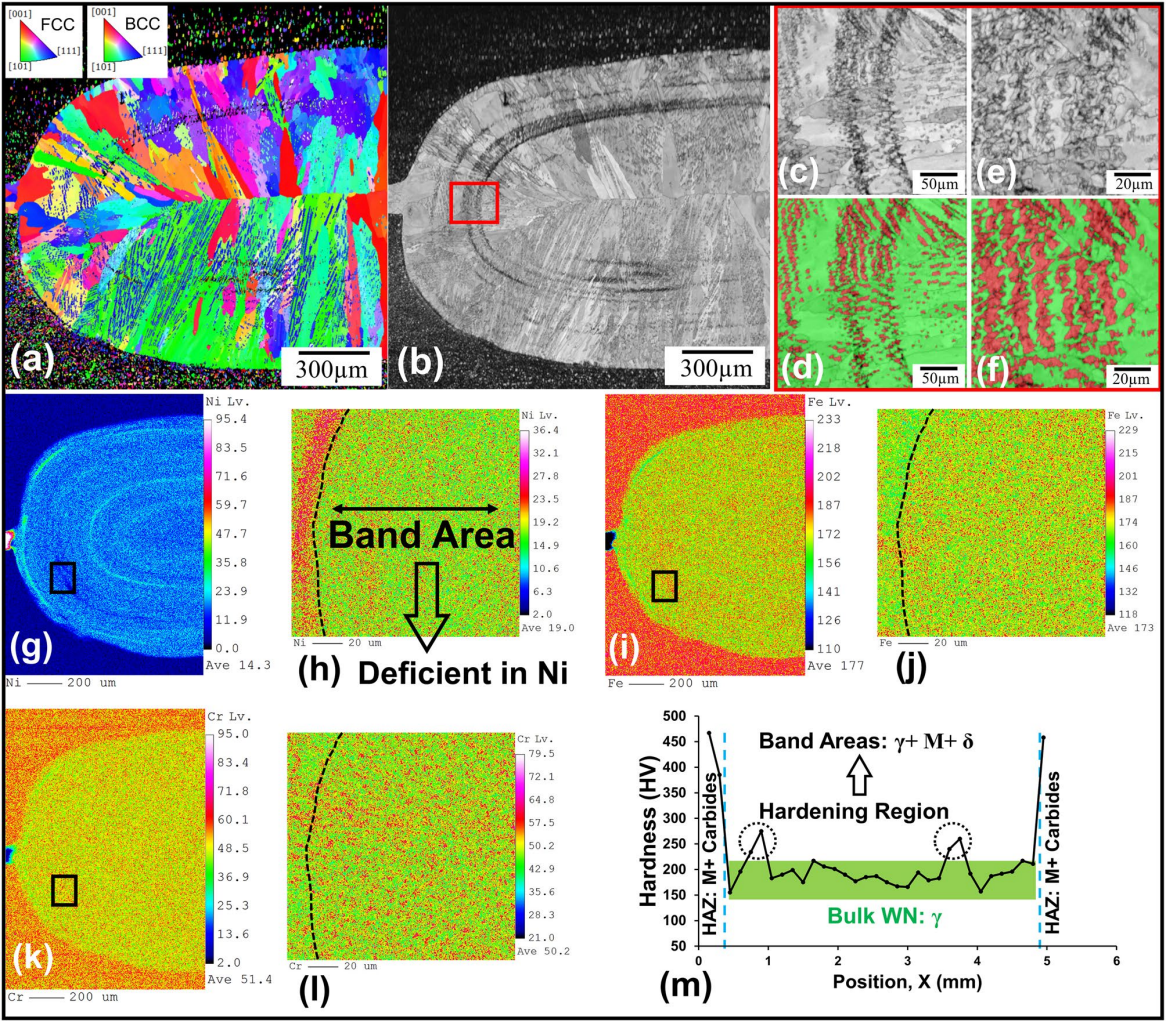
(**a**) EBSD IPF-Z map, (**b**,**c**,**e**) band contrast and (**d**,**f**) phase map analysis of Ni-coated MSS spot weld with red for BCC phase (M/δ) and green for FCC phase (γ), (**g**–**l**) EPMA analysis of a portion of weld nugget in Ni-coated MSS spot weld and higher magnification (black box) for the main elements (Ni, Fe, Cr), and (**m**) hardness profile of Ni-coated MSS spot weld. (M: Martensite, γ: Austenite, δ: Delta-ferrite).

Figure 3a–f shows the images of EBSD analysis of the WN in Ni-coated MSS and its surrounding. As evidenced by the IPF-Z and band contrast EBSD images, the microstructure of the band regions differs from that of the other regions in the WN. Utilizing the phase-map analysis further discerns that the band regions comprise a mix of M/δ and γ phases, while the bulk WN consist of a fully γ microstructure. Figure 3g–l depicts the results of EPMA analysis conducted on a portion of the nugget and higher magnification from band regions. As can be seen, in the band areas, the lack of complete mixing of the BM and the Ni layer coating has occurred, marked by either the absence of Ni or an abundance of Fe in these areas. This distinct chemical composition aligns with the formation of diverse microstructures within the band areas, distinguishing these zones from the rest of the WN. In contrast, the chemical composition remains consistent and uniformly austenitic in the bulk of WN. It is worth mentioning that with the aid of EBSD phase map analysis, the WN contains over than 80% of γ, and the rest being a M/δ mixture. Remarkably, this latter microstructure, signifying a mix of γ and M/δ phases, is primarily localized within the identified band regions. Microhardness results (shown in Fig. 3m) also confirm that band regions have higher hardness (reaching up to 280 HV) over the bulk WN (~ 180–220 HV), corroborating that the microstructure formed in these specific regions (M/δ + γ). The emergence of band zones in the WN is a consequence of a very short time in the ultra-fast RSW process (welding time: 0.24 s), wherein the insufficient time does not permit the complete mixing between the melted Ni coated layer and MSSs.

### Equilibrium and non-equilibrium solidification/solid-state transformations: transitioning solidification mode

**Figure 4:**
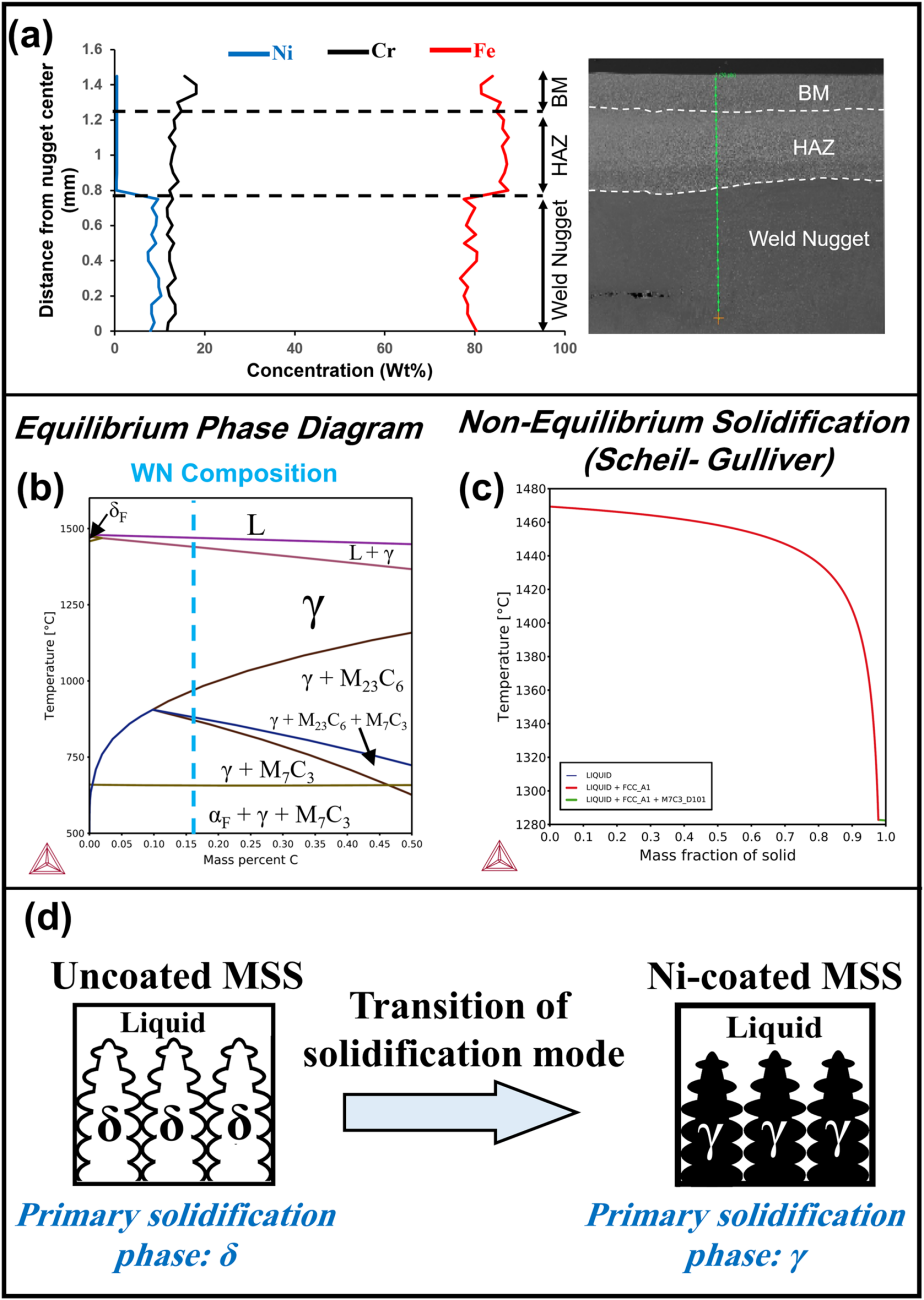
(**a**) Chemical composition profile from nugget center to base metal using EDS line scan in 30 different points, (**b**) pseudo-binary equilibrium phase diagram, and (**c**) Scheil-Gulliver solidification modelling^13^ of the weld nugget of the Ni-coated MSS spot weld, (**d**) Schematic of solidification mode of weld nugget in uncoated and Ni-coated MSS spot welds.

To further explore the microstructures emerging within the WN, a linear EDS scan was taken from 30 different points from the center of the nugget to the BM to determine the chemical composition of the WN. Figure 4a presents the resulting chemical composition profile, detailing the concentrations of the main elements (Ni, Cr, Fe) along this line. The average chemical composition of the WN is Fe-12.41 Cr- 8.75 Ni- 0.1643 C (wt%). It is of note that due to the inability of the EDS and EPMA methods to quantify the carbon, the amount of this element was estimated by the dilution method, as detailed elsewhere^13^. Figure 4b,c illustrates the equilibrium phase and non-equilibrium solidification modelling (i.e. Scheil-Gulliver) diagrams for the chemical composition for the WN obtained from Thermo-Calc software^31^. In the Scheil-Gulliver solidification modelling, the assumption is made that diffusion in the liquid is complete, while there is no diffusion in the solid phase, which is closely resembling the conditions encountered in the RSW process. It should be emphasized that although the cooling rate is very high in the RSW process, considering that the non-equilibrium Scheil-Gulliver modelling only predicts the solidification transformation, here the equilibrium phase diagram is used as a guide to simulate the solid-state transformations. Utilizing both non-equilibrium and equilibrium phase diagrams, the sequence of phase transformations is as follows:

1 $$(\mathrm{Solidification\, path\, from \,Scheil}-\mathrm{Gulliver\, diagram}):\mathrm{ L}\to {\text{L}}+{\upgamma }_{{\text{P}}}\to {\text{L}}+{\left(\upgamma +{{\text{M}}}_{7}{{\text{C}}}_{3}\right)}_{{\text{e}}}$$

2 $$({\text{Solid}}{-}\mathrm{state\, path\, from\, equilibrium\, phase\, diagram}):\upgamma \to\upgamma +{{\text{M}}}_{23}{{\text{C}}}_{6}\to\upgamma +{{\text{M}}}_{7}{{\text{C}}}_{3}+{{\text{M}}}_{23}{{\text{C}}}_{6}\to\upgamma +{{\text{M}}}_{7}{{\text{C}}}_{3}\to\upgamma +\mathrm{\alpha }+{{\text{M}}}_{7}{{\text{C}}}_{3}$$

The primary solidification phase for bare MSS spot weld is δ (schematic shown in Fig. 4d), as detailed elsewhere^13^. However, as evident from Scheil-Gulliver solidification modelling (Eq. (1)), the primary solidification phase is γ for Ni-coated MSS spot weld (schematic shown in Fig. 4d). At the final stages of solidification (f_s_ $\to$ 1), γ and carbides are formed. According to the pseudo-binary equilibrium phase diagram, after solidification, austenite experiences the subsequent solid-state transformations, and finally a microstructure consisting of γ, α and carbide form at room temperature. However, as the EBSD analysis results showed, the bulk WN contains γ microstructure. This observation implies that, due to ultra-fast cooling rate in RSW process (as reported more than 4000 °C/s for 1.5 mm thick MSS RSWs^26^), none of the other solid phases had a chance to form in solid-state diffusion controlled transformations and, as a result, γ remains as a stable phase until room temperature. Notably, the martensite start (M_s_) temperature for this case is around room temperature, indicating that while the cooling conditions necessary for the formation of martensite were met during the RSW process, the WN required further cooling to lower temperatures to initiate the martensitic transformation. Moreover, as observed in EBSD images, the microstructure of the band zones is different from the bulk WN and contains of M/δ in addition to γ. As reported elsewhere^13^, the critical thickness of the Ni interlayer between the 1.5 mm thick MSSs to change the solidification mode is 100 μm. This means that when the interlayer thickness is less than this threshold, the solidification mode is δ, resulting in a dominant martensitic microstructure. On the other hand, when the thickness exceeds 100 μm, the solidification mode is γ, leading to formation of γ in the WN. Therefore, in this critical thickness, a slight deviation from the chemical composition necessary to change the solidification mode causes the appearance of onion-ring structure, which justifies the observed microstructure. Due to similarity of phase transformations, the reader might refer to elsewhere for more microstructural discussions^13^.

### Influence of coating of MSSs with Ni on mechanical performance

**Figure 5:**
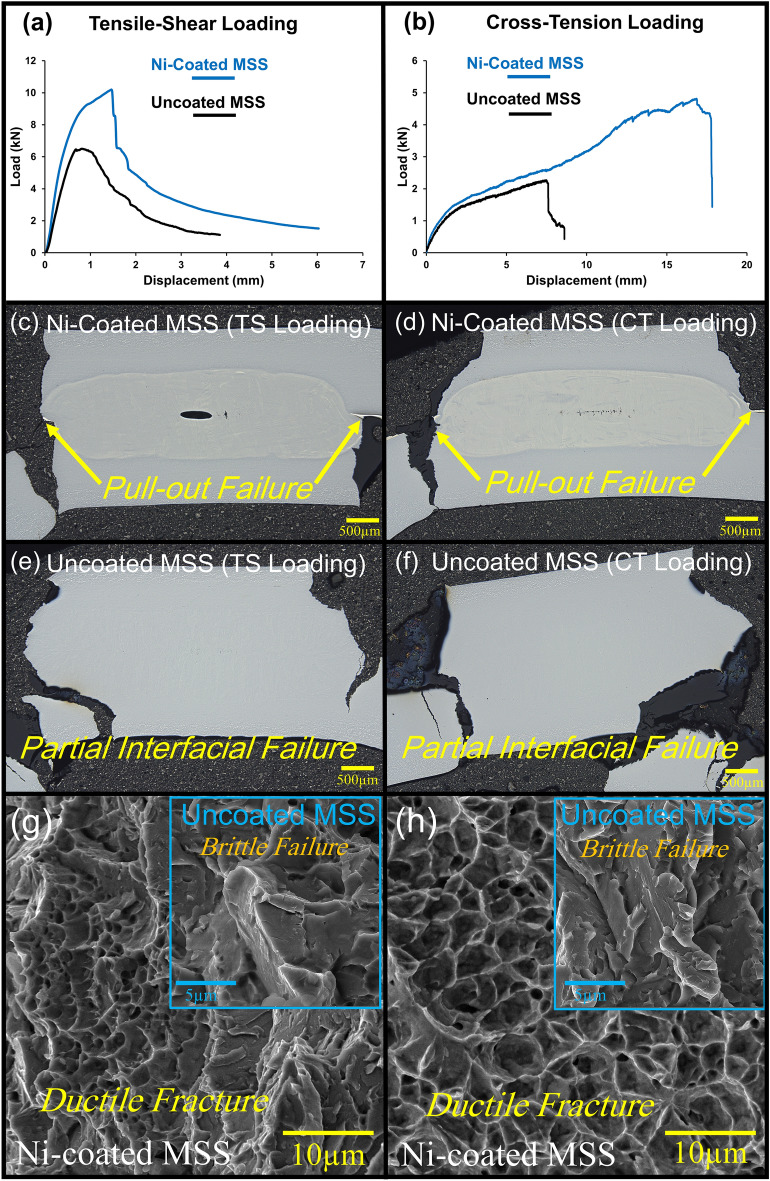
(**a**,**b**) Load–displacement curve of tensile-shear (TS) and cross-tension (CT) loading conditions for Ni-coated MSS and bare MSS spot welds, (**c**,**d**) longitudinal cross-section of Ni-coated MSS spot welds after TS and CT mechanical tests, (**e**,**f**) longitudinal cross-section of uncoated MSS spot welds after TS and CT mechanical tests, and (**g**,**h**) fracture surface of Ni-coated MSS and uncoated MSS (insets) spot welds after TS and CT mechanical tests, respectively.

Figure 5a,b shows the results of the mechanical properties of MSS coated with Ni in TS and CT tests compared with bare MSS spot weld. Clearly, the coating of Ni on MSS sheets through electroplating has led to a significant enhancement in the weldability of these steels, resulting in a substantial improvement in mechanical performance. Specifically, the TS and CT strengths of the Ni-coated MSS spot welds is equal to 10.2 and 5 kN, respectively. These values reflect a notable increase of 57% (1.57 times) and 127% (2.27 times), respectively, compared to conventional bare MSS spot weld. In addition, from energy perspective, Ni-coated MSS spot welds exhibited energy absorption of 9.9 and 52.7 J under TS and CT tests, respectively, reflecting an enhancement of 296% (3.96 times) in TS and 520% (6.2 times) in CT, compared to the non-coated MSS spot weld. As shown in Fig. 5c,d, Ni-coated MSS spot welds experienced pull-out failure (PF) under both TS and CT loading conditions. This qualitative criterion indicates that WN at these spot welds is tough enough to deviate cracks toward thickness direction. In contrast, MSS spot welds without coating exhibited partial interfacial failure (PIF) mode (Fig. 5e,f). It is worth recalling that as shown in Fig. 2a, and also can be observed in Fig. 5c, there is a void at the center of WN. Considering that the failure of the welds occurred outside of the weld nugget (i.e. pull-out failure mode, Fig. 5c,d), it can be concluded that the void did not adversely impact the failure strength of the welds. Furthermore, as shown in Fig. 5g,h, the fracture surface of the Ni-coated MSS spot weld exhibits ductile characteristics, in contrast to the brittle fracture surface observed in the uncoated MSS spot welds (the insets in Fig. 5g,h). Given that the WN size of the bare MSS spot weld (5.8 mm) is larger than that of the Ni-coated MSS (~ 5 mm), and the microstructure of both cases in HAZ is martensite + carbide, it can be concluded that the key factor influencing the mechanical properties of these welds is indeed the microstructure of the WN.

It has been indicated^27,32^ that the mechanical properties of most cases of automotive steels (e.g. DP, HSLA, DQSK, etc.) are influenced by the WN hardness under TS loading. This implies that increasing WN hardness enhances the mechanical properties during TS testing. However, recent studies^11,26^ have highlighted that for AISI420 MSS spot welds, the key factor governing mechanical efficacy is the fracture toughness of the WN during TS testing. Otherwise put, enhancing WN fracture toughness (e.g. achieved via reducing hardness) improves the mechanical response of AISI420 MSS resistance spot welds during TS loading condition.

In addition, in the CT loading, materials with high-hardness values in the weld nugget, e.g. AISI420 MSS which WN hardness exceeds 500HV, the mechanical response of these welds is predominantly controlled by the WN's fracture toughness^11,12,27^. Therefore, the primary strategy to enhance the mechanical performance of AISI420 MSS spot welds under both TS and CT loading conditions is to increase the fracture toughness of the weld nugget. To improve fracture toughness of WN, this study focused on modifying MSS base metals through an electroplating process. This technique aimed to deposit a specific thickness of Ni coating on MSSs, strategically shifting the solidification mode from δ to γ; consequently, this transition led to a predominantly γ microstructure within the WN. As mentioned before and confirmed in other studies^1,8,9,11–13^, uncoated MSSs compose of predominantly brittle martensite in WN. In contrast, as shown in Figs. 3 and 4, the WN in Ni-coated MSS spot welds comprises of mainly tough γ. This tough microstructure confirm the superior mechanical properties and failure mode observed in Ni-coated MSS compared to non-coated MSS spot welds.

It is noteworthy that in both TS and CT tests, the fracture location for Ni-coated MSSs occurs outside the weld nugget (i.e., pull-out fracture mode). Consequently, it can be inferred that the existence of incompletely-mixed bands with M/δ microstructure within the weld nugget does not adversely impact the weld's failure characteristics. This implies that the presence of incompletely-mixed bands does not induce localized brittleness in the weld nugget due to their moderate hardness.

### Final remarks

Previous research findings have highlighted that the most effective strategy for enhancing the toughness of AISI420 MSS spot welds is tailoring the microstructure of the WN towards achieving a predominantly austenitic microstructure. It has been demonstrated that the utilization of a 100 μm Ni interlayer between 1.5 mm thick MSSs promotes the formation of ultra-tough austenitic microstructure in WN^13^. In line with this approach and to realize the full advantage of microstructure engineering and implement it in the industry, a Ni electroplating coating with a thickness of 50 μm was applied on one side of each of the MSS sheets, thereby ensuring a total of 100 μm Ni thickness to be in contact for welding. Given the mechanical properties results obtained in prior work^13^, the Ni-coated MSS has similar mechanical response to the sample with 100 μm thick Ni interlayer, validating the effectiveness of electroplating method in terms of industrial viewpoint.

Considering the results observed in this study, it is possible that reducing the thickness of the coating may result in similar mechanical properties as those found in the sample with Ni interlayer of identical thickness. As indicated in a previous study^13^, although a Ni interlayer below 100 μm was not successful in changing the solidification mode from δ to γ, inserting of a 50 μm interlayer enhanced TS mechanical properties and marginally satisfied CT properties. Thus, this study suggests there may be a potential to further decrease this thickness to a more cost-effective 25 μm Ni coating on each MSS sheet (providing a total of 50 μm in contact). Thus, if one is aiming to achieve sufficient mechanical properties to pass the minimum strength standard, a 25 μm coating might be a good choice. Moreover, recalling that the welding current in this study was 9 kA. Thus, employing a higher welding current of a thinner coating (less than 50 μm on one side of MSSs) could potentially alter the solidification mode due to a reduced dilution ratio. This possibility presents an interesting research study for further exploration in subsequent investigations. Finally, it should be kept in mind that the transition of solidification mode from ferrite (BCC) to austenite (FCC) can increase the susceptibility to hot tearing. This implies that the formation of γ microstructure in the weld nugget is a trade-off between the development of a microstructure with high fracture toughness and the formation of defects like hot tears. By increasing the thickness of the nickel layer coating and so, increasing the austenite, the possibility of hot crack formation increases and vice versa. In this work, as mentioned, with a 50 μm coating, pull-out failure was achieved under loading conditions and the pores did not have an adverse effect on the fracture strength. Therefore, when designing nickel coatings for MSSs, there should be a balance between achieving a microstructure with superior fracture toughness and mitigating the occurrence of defects such as hot tears within the weld nugget. Last but not least, it is noteworthy that the RSW of galvanized steels has gained attention in the automotive industry in recent years. However, issues related to liquid metal embrittlement (LME) have limited the utilization of these sheets in certain applications^33–36^. The exceptional outcomes observed with spot welding of Ni coating on MSSs in this novel study have the potential to open new opportunities for the deployment of Ni-coated AISI420 MSSs with an optimum thickness of Ni coating in the automotive industry necessitating both strength and corrosion resistance properties.

## Conclusion

Resistance spot welding (RSW) of martensitic stainless steels (MSSs) leads to joints with low fracture toughness due to the formation of a brittle martensitic microstructure in both weld nugget (WN) and heat-affected zones. This brittle microstructure at the joint area of MSS spot welds triggers premature failure in tensile-shear (TS) and cross-tension (CT) loading conditions, limiting their applications in the automotive industry. The most effective strategy to enhance fracture toughness of MSS spot welds is microstructure engineering of the weld nugget (WN) towards achieving a predominantly tough austenitic microstructure. This work aimed to enhance the fracture toughness of MSSs spot welds by employing a novel approach: electroplating a 50 μm thick layer of Ni onto 1.5 mm thick MSS sheets. With the aid of the equilibrium and non-equilibrium Scheil-Gulliver modelling, it was shown that the coating of MSSs with Ni altered the solidification mode from δ (ferrite) to γ (austenite) within the weld nugget, resulting in a predominantly tough austenitic microstructure. Moreover, the partial mixing of the Ni coating with the MSS base metals produced distinct band structures exhibiting diverse microstructures (including M, δ, and γ phases), contrasting with the fully austenitic microstructure typically found in the bulk WN. The results of this study demonstrated a significant enhancement in the mechanical properties of Ni-coated MSS spot welds when compared to their non-coated welds. Specifically, the TS and CT strengths of Ni-coated MSS spot welds increased by 1.57 times and 2.27 times, respectively, in comparison to bare MSS spot welds. Additionally, from the energy viewpoint, the energy absorption during TS and CT tests improved by 3.96 times and 6.2 times, respectively, showcasing the remarkable improvement in weldability. Moreover, the Ni-coated MSS spot welds exhibited desirable pull-out failure (PF) behavior under both TS and CT loading conditions, contrasting with the partial interfacial failure (PIF) mode observed in bare MSS spot welds. These improvements can have profound implications for the automotive industry, where the demand for high-strength, energy absorption and corrosion-resistant materials is substantial. The exceptional quantitative and qualitative outcomes observed in this study have the potential to provide the industry with MSS sheets with Ni coating that offer both high strength/energy and superior corrosion resistance properties, making them a promising alternative for automotive industry.

## Data Availability

The data will be made available upon request. Correspondence and requests for materials should be addressed to H.A. and M.P.
